# Identification of Soil Microbes Capable of Utilizing Cellobiosan

**DOI:** 10.1371/journal.pone.0149336

**Published:** 2016-02-12

**Authors:** Jieni Lian, Jinlyung Choi, Yee Shiean Tan, Adina Howe, Zhiyou Wen, Laura R. Jarboe

**Affiliations:** 1 Bioeconomy Institute, Iowa State University, Ames, Iowa, United States of America; 2 Department of Agricultural and Biosystems Engineering, Iowa State University, Ames, Iowa, United States of America; 3 Department of Chemical and Biological Engineering, Iowa State University, Ames, Iowa, United States of America; 4 Department of Food Science and Human Nutrition, Iowa State University, Ames, Iowa, United States of America; West Chester University of Pennsylvania, UNITED STATES

## Abstract

Approximately 100 million tons of anhydrosugars, such as levoglucosan and cellobiosan, are produced through biomass burning every year. These sugars are also produced through fast pyrolysis, the controlled thermal depolymerization of biomass. While the microbial pathways associated with levoglucosan utilization have been characterized, there is little known about cellobiosan utilization. Here we describe the isolation and characterization of six cellobiosan-utilizing microbes from soil samples. Each of these organisms is capable of using both cellobiosan and levoglucosan as sole carbon source, though both minimal and rich media cellobiosan supported significantly higher biomass production than levoglucosan. Ribosomal sequencing was used to identify the closest reported match for these organisms: *Sphingobacterium multivorum*, *Acinetobacter oleivorans* JC3-1, *Enterobacter* sp SJZ-6, and *Microbacterium* sps FXJ8.207 and 203 and a fungal species *Cryptococcus* sp. The commercially-acquired *Enterobacter cloacae* DSM 16657 showed growth on levoglucosan and cellobiosan, supporting our isolate identification. Analysis of an existing database of 16S rRNA amplicons from Iowa soil samples confirmed the representation of our five bacterial isolates and four previously-reported levoglucosan-utilizing bacterial isolates in other soil samples and provided insight into their population distributions. Phylogenetic analysis of the 16S rRNA and 18S rRNA of strains previously reported to utilize levoglucosan and our newfound isolates showed that the organisms isolated in this study are distinct from previously described anhydrosugar-utilizing microbial species.

## Introduction

**Fig 1:**
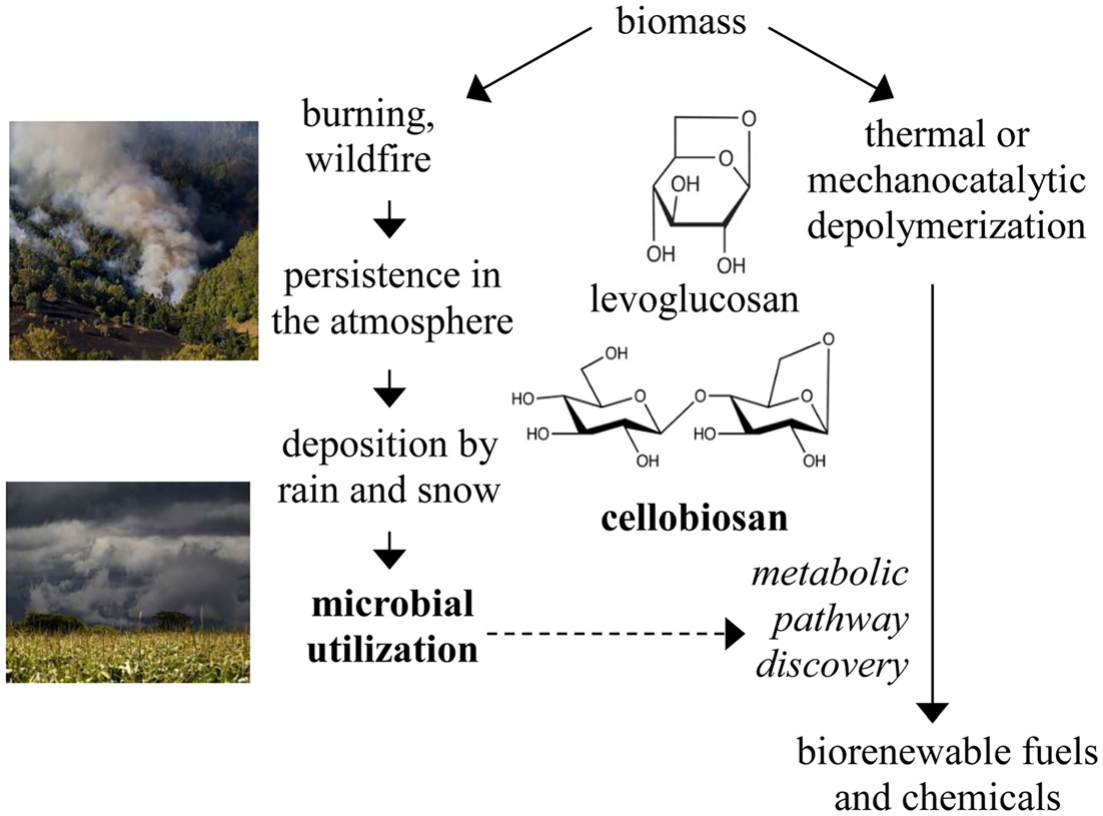
Overview of the anhydrosugar cycle and its relevance to the production of biorenewable fuels and chemicals. Images are from creative commons.

Anhydrosugars, such as levoglucosan, cellobiosan, mannosan, galactosan, levogalactosan, and levomannosan, are produced from the burning of biomass [1, 2] and have been measured in wildfire smoke at a concentration of 24 mg anhydrosugars per g of organic carbon [3]. These anhydrosugars have also been detected in rainwater [4], presumably resulting in the cycling of these atmospheric compounds to the soil. Using the estimate that approximately 4 billion metric tons of carbon are released by biomass burning every year [5], we estimate that 90 million metric tons of anhydrosugars are produced every year, representing a substantial and under characterized portion of the global carbon cycle. A biomass/atmosphere/soil anhydrosugar cycle (Fig 1) is consistent with the detection of anhydrosugars in such diverse locations as soils, aerosols, snow pits and even human urine [6–8].

In addition to production through typical biomass burning processes, anhydrosugars are also produced during the controlled thermochemical depolymerization of biomass known as fast pyrolysis [9]. While levoglucosan is the most well-characterized anhydrosugar product of biomass pyrolysis, cellobiosan is also present in the pyrolysis product [9, 10]. Specifically, up to 12 wt% of pyrolyzed cellulose has been recovered as cellobiosan [11] and in some cases, cellobiosan is present in the pyrolysis product at levels up to 30 wt% of the levoglucosan content [12, 13]. It has even been proposed that cellobiosan is the primary product of fast pyrolysis [14]. It should be noted that cellobiosan can be hydrolyzed to produce one molecule of levoglucosan and one molecule of glucose [12].

These biomass-derived anhydrosugars are an attractive substrate for the production of biorenewable fuels and chemicals [15]. While standard industrial organisms such as *Escherichia coli* are unable to metabolize levoglucosan [16] and, presumably, other anhydrosugars, studies have reported microbial degradation of anhydrosugars in soil [17]. This microbial activity is an important part of the anhydrosugar cycle and identification and characterization of the associated enzymes and pathways may enable implementation of these pathways in other organisms.

While we are interested in understanding the metabolic pathways associated with utilization of all anhydrosugars, such information has been reported only for levoglucosan. Specifically, microbial utilization of levoglucosan has been described through levoglucosan kinase [18–23] and levoglucosan dehydrogenase [24]. Identification and characterization of these pathways has enabled the engineering of industrially relevant organisms, such as ethanologenic *E*. *coli*, for levoglucosan utilization [16]. This demonstrates that identification of organisms that are capable of metabolizing less-characterized anhydrosugars, such as cellobiosan, is useful to understanding the anhydrosugar cycle and to the eventual engineering of microbes for utilization of a range of anhydrosugars.

The goal of this study is to find and identify microorganisms capable of utilizing cellobiosan. Given that anhydrosugars are transferred from the atmosphere to the soil [4], we used soil samples as a possible source of cellobiosan-utilizing organisms. Identification of these organisms will guide future characterization of cellobiosan metabolic pathways.

## Materials and Methods

### Chemicals

The anhydrosugars levoglucosan (C_6_H_10_O_5_, CAS Number, 498-07-7, 6-anhydro-β-d-glucopyranose) and cellobiosan (C_12_H_20_O_10_, CAS Number, 35405-71-1, β-d-glucopyranosyl-(1–4)-1,6-anhydro-d-glucopyranose or 1,6-anhydro-β-cellobiose) were obtained from Carbonsynth (San Francisco, USA). All other chemicals were purchased from Fisher Scientific.

### Isolate collection

Soil was collected randomly at a depth of less than 15 cm in Ames, Iowa on private land owned by the corresponding author. This soil had been used over the previous four years to grow tomatoes and sunflowers, with occasional application of commercial herbicides and enrichment with wood-fire ashes. Five grams of this soil was suspended in 50 ml of sterile E-pure water and mixed by manual shaking at room temperature for 5 mins. The aqueous phase was separated by centrifugation at 3,000g, room temperature, for 10 mins. The solid phase (soil) and aqueous soil extract were each spread onto M9-cellobiosan mineral agar plates (20 g L^-1^ cellobiosan, 12.8 g L^-1^ Na_2_HPO_4_∙7H_2_O, 3 g L^-1^ KH_2_PO_4_, 0.5 g L^-1^ NaCl, 1.0 g L^-1^ NH_4_Cl, 0.24 g L^-1^ MgSO_4_, 0.01 g L^-1^ CaCl_2,_ 12 g L^-1^ agar, pH 6.0). The agar plates were cultured at 30°C for 48 hours.

### Isolate identification and characterization

Single colonies were selected and further isolated on LB plates and then distinguished by morphologies signatures, such as the shape, surface and color of the colony. *Enterobacter* DSM16657 (Deutsche Sammlung von Mikroorganismen und Zellkulturen, Germany) was maintained on LB agar plate. DSM16657 and our isolates were characterized by culturing in liquid LB or mineral M9 media with 2.0 wt% levoglucosan or cellobiosan in shake flasks at 200 rpm, 30°C for 24 hours. Both media types had an initial pH of 6.0. Growth was monitored by absorbance at 550 nm (Thermo Spectronic 20 Genesys, US).

DNA was extracted from isolates, and 16S rRNA gene sequences were amplified with PCR. For the isolates S2, S3, S4 and S5, the 16S rRNA sequences were amplified using oligonucleotide primers: 27F AGAGTTTGATCMTGGCTCAG, and 1492R CGGTTACCTTGTTACGACTT synthesized by Integrated DNA Technologies, USA. PCR amplification reactions used Q5 High-Fidelity DNA Polymerase (New England Biolabs, US) with the denaturing temperature 98°C for 30 seconds, annealing temperature 55°C for 20 seconds and extension temperature 72°C for 1 minute. The PCR products were purified by QIAquick PCR Purification Kit (Qiagen, US), quantified by NanoDrop (Thermo Fisher Scientific, USA), diluted to a concentration of 2.5 ng/100 bases/μl, and sequenced at the Iowa State University DNA Facility. The resulting 16S and 18S rRNA sequences for all isolates were compared to the existing sequences through the National Center for Biotechnology Information (NCBI) BLAST database.

#### 2.4 16S rRNA gene amplicon sequencing and phylogenetic analysis

The paired-end 16S rRNA gene sequences for each strain were assembled manually followed by alignment using CLUSTALW [25]. The isolate 16S rRNA gene amplicon sequences were also compared to environmental 16S rRNA gene amplicon sequences originating from soils at the Comparison of Biofuel Systems (COBS) of Iowa State University [26, 27]. The COBS sequencing database is available at MG-RAST, project 2592 [28].

Additionally, isolate 16S rRNA sequences were compared to the 16S and 18S rRNA sequences of nine species previously reported either to utilize levoglucosan or encode levoglucosan kinase [20, 21, 29], obtained from the NCBI RefSeq database [30] (Table 1). The 16S rRNA gene sequences of isolates were also compared to all available 16S rRNA gene sequences contained within the Ribosomal Database Project (RdP, Release 11) [31] using the Infernal aligner version 1.1.1 [32]. Selected aligned 16S rRNA gene sequences from well-characterized type strains were used with genes from isolates to construct a phylogenetic tree. The tree was built using the Maximum Likelihood based on the Jukes-Cantor model by Fasttree (version 2.1.8) using default parameters [33].

**Table 1:** 16S rRNA gene-based identification of soil isolates and population analysis of 16S rRNA gene for five cellobiosan-utilizing bacterial isolates and four levoglucosan-utilizing bacterial isolates based on sequences of Iowa COBS soil microbial community. Isolates S1, S2, S3, S4, S5, and F6 utilize cellobiosan. Isolates 1, 2, 3 and 4 utilization levogluocsan. OTU: Operational taxonomic unit, here is defined as genes sharing 97% sequence similarity in the COBS dataset. Abundance: the relative abundance of OTU within COBS dataset.

NCBI BLAST (nr)					COBS				
Closest Match		Identity (%)	Length of match	Gene ID	Identity (%)	Length of match	Abundance	OTU	Relative abundance
**S1**	*Enterobacter* sp SJZ-6	99	604/605	dbj|LC014955.1|	99	127/128	360	922761	0.0110
**S2**	*Sphingobacterium multivorum*	97	1316/1359	dbj|AB680844.1|	99	252/253	50	891031	0.0015
**S3**	*Acinetobacter oleivorans* JC3-1	98	1352/1385	gb|KM983423.1|	99	251/253	70	889025	0.0021
**S4**	*Microbacterium* sp FXJ8.207	98	1353/1383	gb|KM507662.1|	98	270/277	10	1045797	0.0003
**S5**	*Microbacterium* sp FXJ8.203	98	1302/1330	gb|JQ012996.1|	98	269/277	10	1045797	0.0003
**F6**	*Cryptococcus* sp	95	686/722	gb|KM587000.1|	N/A	N/A	N/A	N/A	N/A
**1**	*Bacillus horikoshii*	97	1361/1396	gb|KJ534599.1|	100	253/253	22	591482	0.0037
**2**	*Bacillus korlensis* 1	95	1336/1402	gb|KC443095.1|	98	244/249	158	42013	0.38
**3**	*Bacillus korlensis* 2	96	1344/1407	gb|KC443095.1|	94	236/250	158	42013	0.38
**4**	*Bacillus* sp 5138	97	1358/1400	gb|KC236668.1|	98	250/254	302	848816	0.036

Sequences acquired here are available for download on NCBI (pending).

## Results

### Identification of soil isolates capable of cellobiosan utilization

**Fig 2:**
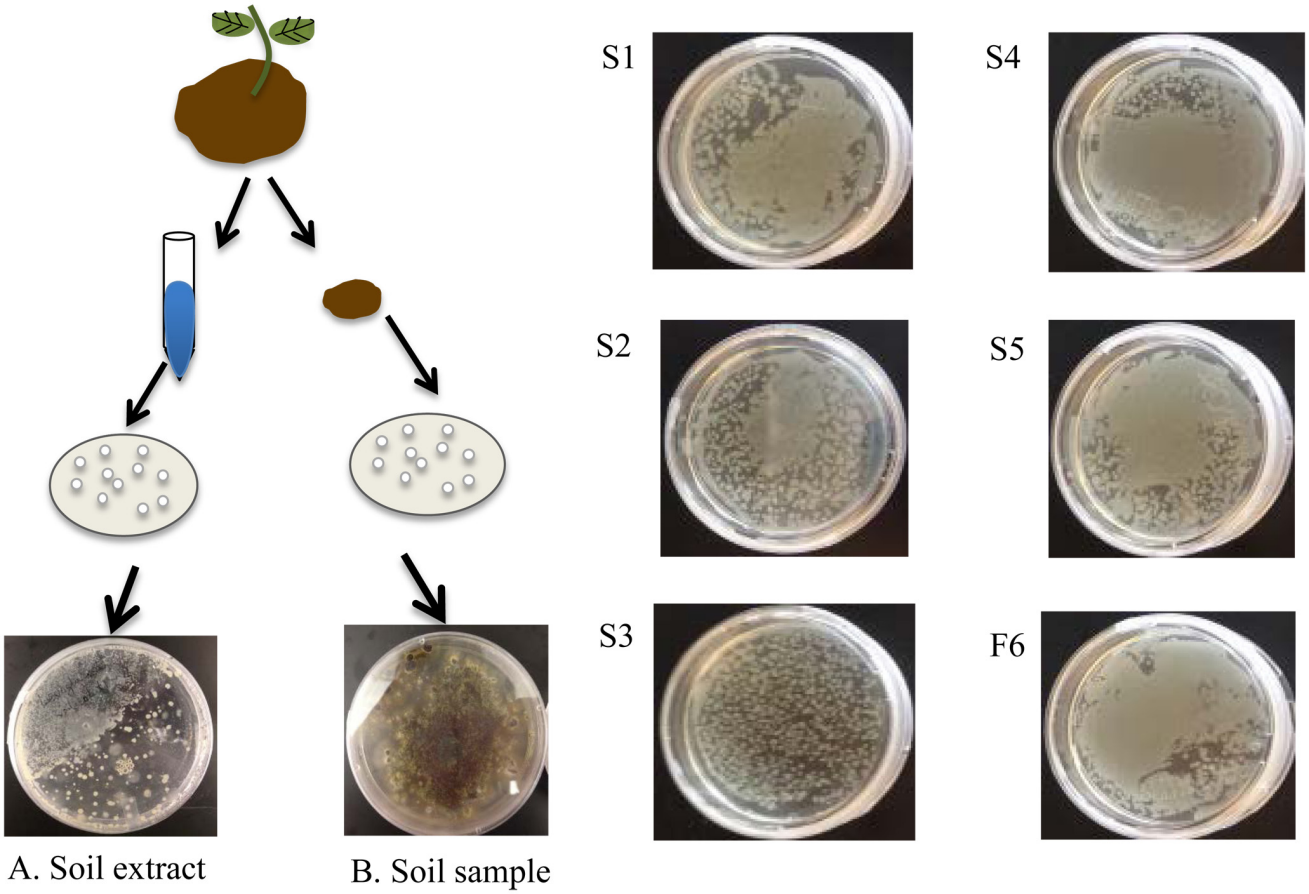
Isolation of microbes capable of using cellobiosan as sole carbon source. (A) Aqueous soil extract, (B) Soil. Pure cultures of S1-S5 and F6. All plates consisted of M9 with 2.0 wt% cellobiosan and were grown at 30°C for 24 hours.

Six soil organisms capable of utilizing cellobiosan were isolated by growth on minimal media plates containing cellobiosan as the sole carbon source. Colonies were isolated from both the solid soil sample and the aqueous soil extract (Fig 2). The five bacterial isolates were designated S1–S5 and the one fungal isolate was designated F6. Isolates S1-S4 were obtained from the solid soil sample and S5 and F6 were obtained from the aqueous soil extract.

The 16S and 18S rRNA gene sequences of our isolates were obtained and the closest matching species with the NCBI nr database were identified (Table 1). For isolates S1-S5, the following species were identified as the closest matches: *Enterobacter* sp SJZ-6, *Sphingobacterium multivorum*, *Acinetobacter oleivorans* JC3-1 and *Microbacterium* sps FXJ8.207 and 203. Each of these matches had 97–99% similarity. A 95% match to a *Cryptococcus* sp was identified for fungal isolate F6.

**Fig 3:**
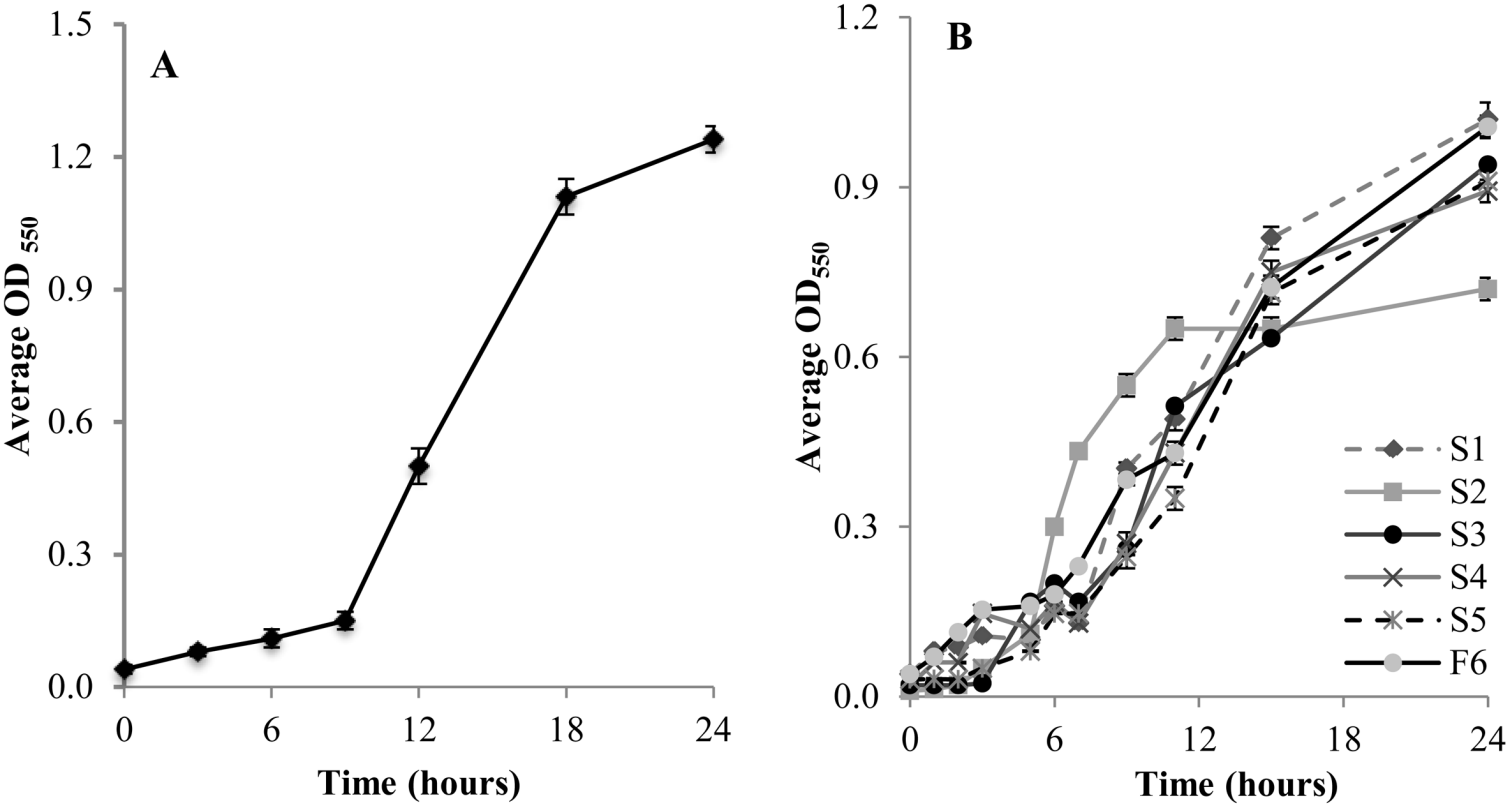
Microbial growth on cellobiosan. (A) *Enterobacter* DSM16657, (B) our soil isolates on M9 minimal medium with 2.0 wt% cellobiosan at 30°C, 200 rpm for 24 hours. Data is the average of 3 biological replicates, with error bars showing one standard deviation.

To validate these sequencing results, we obtained and tested a commercially-available organism with 16S rRNA gene high sequence similarity to one of our isolates. Specifically, we obtained *Enterobacter cloacae* DSM 16657 as a counterpart to our isolate S1, which is a 99% identity match with *Enterobacter* sp SJZ-6 (Table 1). Consistent with our identification of isolate S1 as an *Enterobacter* species, *E*. *cloacae* DSM16657 was able to use cellobiosan as sole carbon source (Fig 3A).

### Characterization of anhydrosugar utilization

Each of our six isolates was further characterized by culturing in liquid minimal M9 media containing 2.0 wt% cellobiosan (Fig 3B). Isolate S2 reached a significantly higher (P≤0.05) optical density than the other organisms in the 6–12 hour time points, but the final optical density at 24 hours was decreased relative to the other species. The growth of the other 5 isolates was similar to each other. The model bacteria *E*. *coli* KO11 and DH5alpha were used as negative controls and showed no growth in M9 medium containing 2.0 wt% cellobiosan (*data not shown*). Since cellobiosan is the only carbon source available in this M9 medium, these results demonstrate that these *E*. *coli* strains cannot utilize cellobiosan as a sole carbon source.

**Fig 4:**
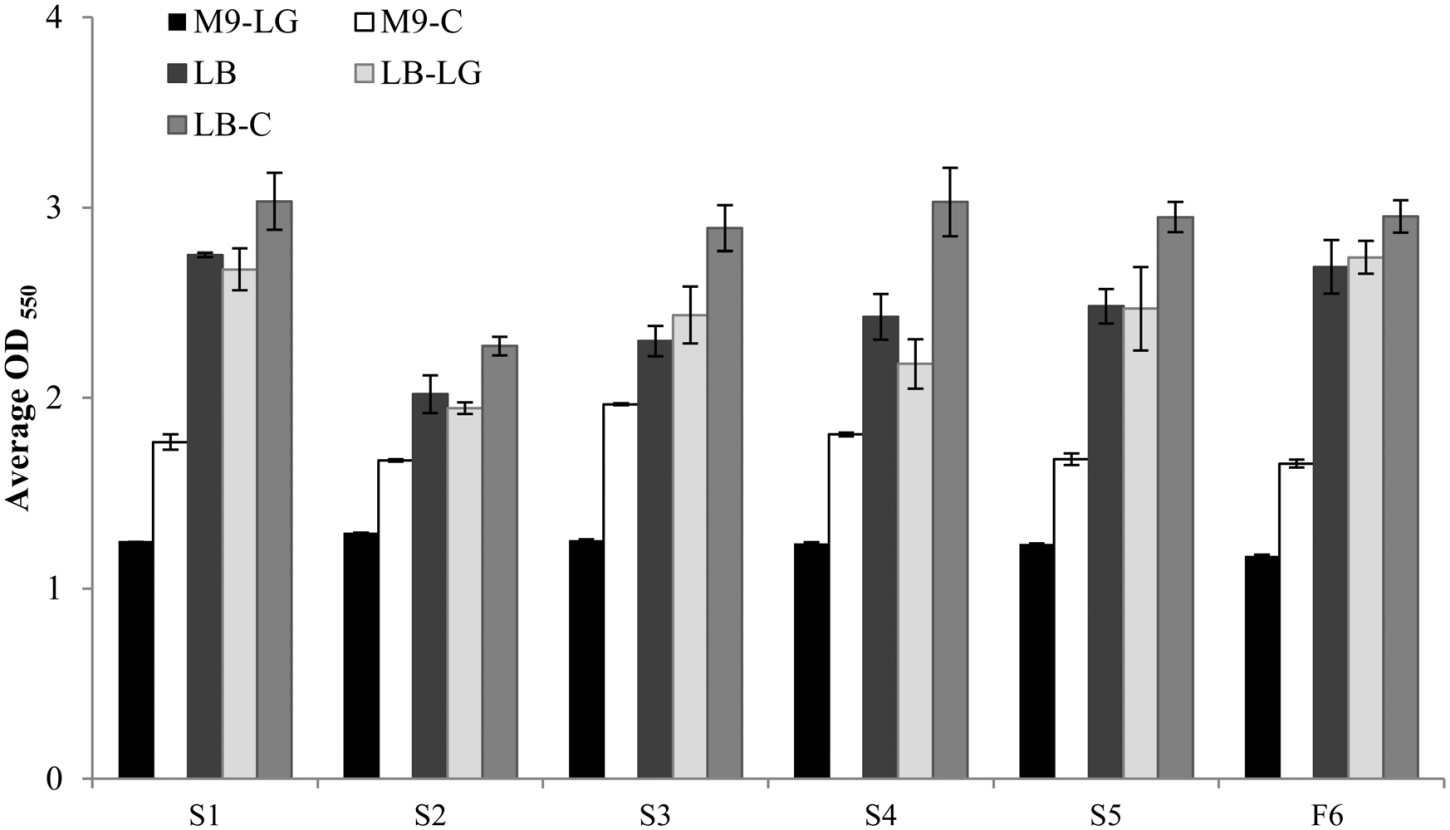
Growth of our isolates on levoglucosan and cellobiosan. Growth of isolates in M9 with 2.0 wt% levoglucosan (M9-LG), M9 with 2.0 wt% cellobiosan (M9-C), LB with no supplemental carbon (LB), LB with 2.0 wt% levoglucosan (LB-LG), and LB with 2.0 wt% cellobiosan (LB-C). Note that M9 is a mineral salts media and a supplemental carbon source must be provided to support microbial growth. Thus, M9 with no supplemental carbon is not feasible. Isolates were grown for 24 hours at 30°C, 200 rpm. Data is the average of three biological replicates and error bars indicate the standard deviation. P values were < 0.01 for all organisms in M9-LG versus M9-C and < 0.05 for all organisms in LB-LG vs. LB-C.

In addition to identifying these 6 isolates as capable of utilizing cellobiosan as sole carbon source, we also compared their growth on minimal (M9) and rich (LB) medium supplemented with levoglucosan (Fig 4). These results showed that all of the cellobiosan-utilizing organisms isolated in our study were also capable of using levoglucosan as sole carbon source. However, in both minimal media and rich media, all organisms showed significantly higher (P≤0.05) OD_550_ values on 2.0 wt% cellobiosan than 2.0 wt% levoglucosan. Note that 2.0 wt% cellobiosan and 2.0 wt% levoglucosan both supply 0.89 wt% carbon.

### Phylogenetic analysis

**Fig 5:**
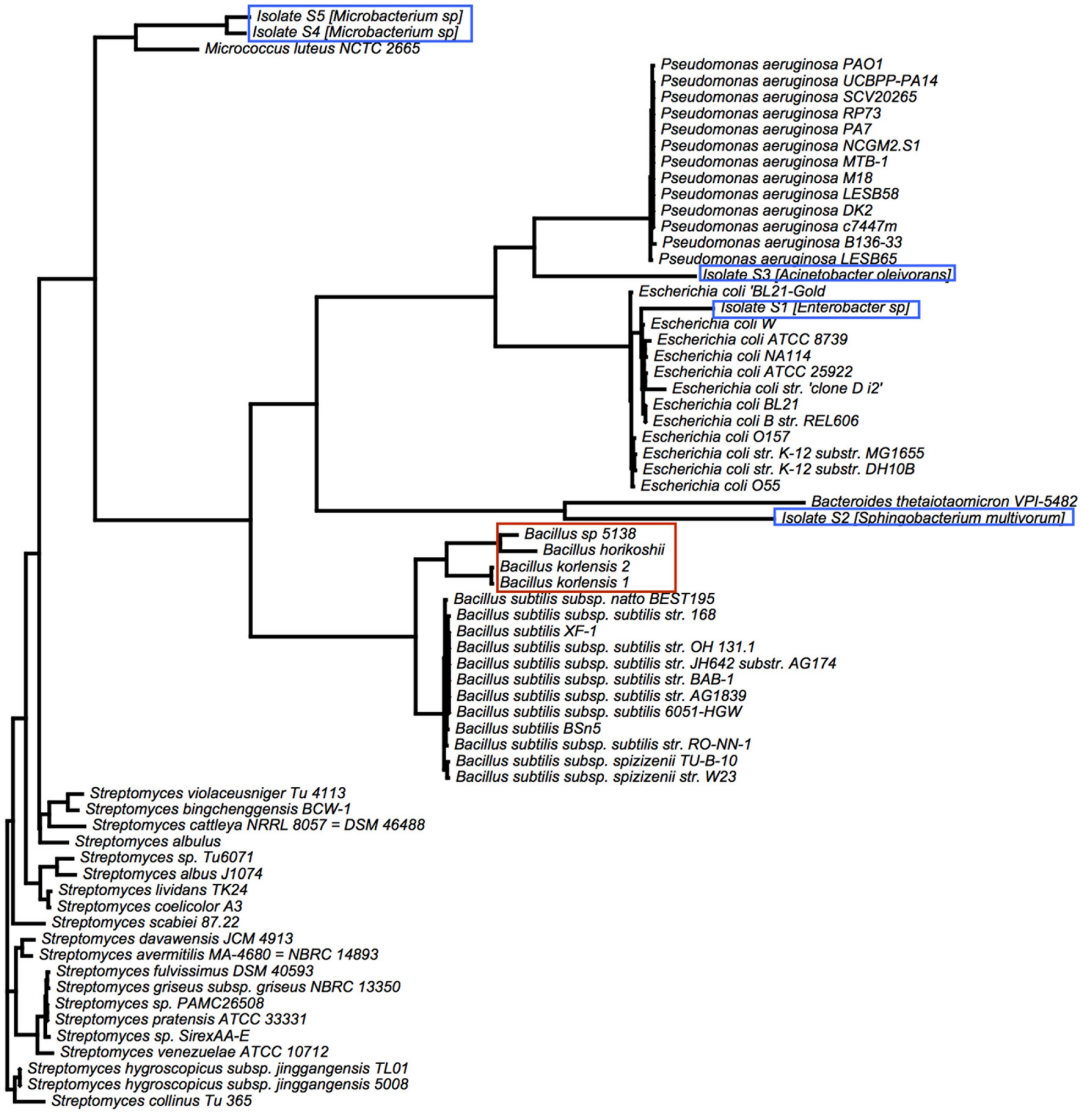
Phylogenetic analysis. A maximum likelihood reconstruction of the phylogenetic analysis of 16S rRNA genes of our isolates (blue boxes) and of selected type strains. Isolates are more diverse than previously reported levogluosan-utilizing isolates (red box). The closest relative to isolates by 16S rRNA gene similarity in the RDP database is shown in parentheses.

For a better understanding of where these isolates fit within the context of known microbial species, we performed a phylogenetic analysis of our isolates along with well-characterized type strains (Fig 5). Previously reported levoglucosan-utilizing isolates have been associated with *Bacillus*. With the exception of isolate S2 this phylogenetic analysis reveals that our isolates are more diverse. Isolates S4 and S5 are most similar to *Micrococcus*, isolates S1, S2, and S3 are more closely related to *Pseudomonas*, *Bacteriodetes*, and *Escherichia* respectively.

### Comparison of isolates to soil microbial communities

The isolates described in this study were collected from one soil sample from a single sampling location. In order to evaluate their presence in other soil samples, we used a publicly available dataset of 16S rRNA gene amplicons obtained from Iowa soils located within 20 miles of our sampling site. The rRNA gene sequence of each of our isolates matched sequences in this database with up to 97–99% similarity. These results confirm that each of our five microbial isolates our similar to the 16S rrNA genes of other native soil microorganisms albeit not sharing exact sequence identity.

Using the comparison to available soil amplicons, we also estimated the abundance of phylogenetically similar (>97% 16S rRNA gene sequence similarity) species in the soil. This analysis was performed by comparing the 16S rRNA gene V4/V5 region of each of our isolates to an existing database of 16S rRNA amplicons from soil samples (COBS). All amplicon sequences were clustered into operational taxonomic units (OTU) that were defined groups of sequences that shared >97% similarity. The abundance of each OTU in the COBS database was calculated as its relative abundance in the COBS dataset. Our isolate sequences were associated with the COBS OTU that share the highest sequence similarity, allowing us to estimate the abundance of similar species within the soil.

The resulting abundance of our isolate amplicons ranges from 0.0003–0.0110% of all reads (Table 2). The COBS database contains 65,823 distinct amplicons and seuqences similar to our isolates are of population rank between 1,300 and 14,000. Sequences similar to isolate S1, *Enterobacter* sp SJZ-6, showed the highest abundance of any of our isolates. These results demonstrate that amplicons similar to those obtained from our isolates are present in other soil-associated amplicon datasets.

**Table 2:** Strains within the NCBI nr database sharing similarity to the isolates studied here. Strains were selected based on their reported ability to utilize levoglucosan. *Bacillus horikoshii*, *Bacillus korlensis* 1 and 2, and *Bacillus* sp 5138, were originally named bacterium levoglucosan 1, 2, 8, and 10, respectively, and were putatively identified in this work (Table 1).

Organism Name	Source	GenBank ID (NCBI)	Levoglucosan utilization	Cellobiosan utilization	Reference
*Bacillus horikoshii*	16S rRNA	EU661929.1	positive	N/A	NCBI
*Bacillus korlensis* 1	16S rRNA	EU661930.1	positive	N/A	NCBI
*Bacillus korlensis* 2	16S rRNA	EU661931.1	positive	N/A	NCBI
*Bacillus* sp 5138	16S rRNA	EU661932.1	positive	N/A	NCBI
*Enterobacter sp* (S1)	16S rRNA	N/A	positive	positive	This study
*Sphingobacterium multivorum* (S2)	16S rRNA	N/A	positive	positive	This study
*Acinetobacter oleivorans* (S3)	16S rRNA	N/A	positive	positive	This study
*Microbacterium sp* (S4)	16S rRNA	N/A	positive	positive	This study
*Microbacterium sp* (S5)	16S rRNA	N/A	positive	positive	This study
*Penicilium sp* HX-2006g	18S rRNA	DQ333283.1	positive	N/A	[20]
*Alternaria sp* HX2006h	18S rRNA	DQ333284.1	positive	N/A	[20]
*Aspergillus sp* HX2006f	18S rRNA	DQ333282.1	positive	N/A	[21]
*Cryptococcus sp* (F6)	18S rRNA	N/A	positive	positive	This study
*Lipomyces starkeyi*	18S rRNA	JQ698932.1	positive	N/A	[23]
*Rhodosporidium toruloides*	18S rRNA	DQ647614.2	positive	N/A	[29]

## Discussion

Cellobiosan is an important part of the global carbon cycle and is also relevant to the deconstruction of biomass to produce biorenewable fuels and chemicals. Here we provide the first report of microbial utilization of cellobiosan. The results presented here contribute to our understanding of cellobiosan metabolism, though additional studies are required to identify the biological pathways enabling cellobiosan utilization.

Various fungal species have previously been reported to utilize levoglucosan, including species of *Penicilium* [20], *Alternaria* [20], *Aspergillus* [21], *Lipomyces* [23] and *Rhodosporidium* [29] (Table 2). However, to the best of our knowledge, previous reports of bacterial species capable of utilizing levoglucosan were limited to a description of soil isolates that were not identified in the original study [20]. Here we have tentatively identified these previously-described bacterial species via their previously published 16S rRNA. Our characterization and putative identification of cellobiosan-utilization soil isolates also add five more microbes to the known set of bacterial species able to metabolize levogluocsan.

We observed that cellobiosan always supported a significantly higher amount of biomass production for each of these six organisms relative to levoglucosan; this suggests that there may be overflow metabolism during levoglucosan utilization (Fig 4). Overflow metabolism is well characterized for glucose relative to other carbon sources [34]. Essentially, rapid sugar consumption leads to the excretion of carbon in the form of organic acids instead of its incorporation into biomass. It is plausible that levoglucosan is causing overflow metabolism in these organisms relative to cellobiosan.

We also observed that an isolate that is closely related to *S*. *multivorum* grew markedly faster than the other five organisms in minimal media cultures containing cellobiosan as the sole carbon course, though the final amount of biomass produced was lower than the other five organisms (Fig 3). *S*. *multivorum* has been identified in a variety of clinical, environmental and industrial sampling studies [35–37], but to the best of our knowledge, no comparative growth studies have been described in the literature. Thus, it is not clear if this fast growth and low biomass production is specific to cellobiosan or if this is a general hallmark of this organism.

Some of the organisms characterized in this study have previously been associated with industrially-promising metabolic behavior. For example, *S*. *multivorum* has been reported to produce carotenoids, fatty acids and carbolic acids [38]. Therefore, this organism may be an interesting starting point for the microbial utilization of anhydrosugars for the fermentative production of a variety of valuable products.

Phylogenetic analysis of the rRNA sequences of known anhydrosugar-utilizing organisms (Fig 5) highlights the diversity among this under-characterized group of organisms. While our fungal isolate F6 is closely related to the previously-characterized *Cryptococcus*, the five bacterial species isolated here form a distinct cluster relative to the four previously-described organisms.

Comparison of the ribosomal RNA sequences obtained both from our isolates and from previously-characterized organisms showed that organisms closely related to the isolates exist in soil samples other than those acquired here, and thus are possibly widespread in nature. This work is an important first step in the identification of enzymes and pathways enabling cellobiosan utilization. This highlights the importance of turning to under-characterized microbial communities, such as those found in soil, as a source of novel biological activity.
